# Adoption of Sodium Reduction Strategies in Small and Rural Hospitals, Illinois, 2012

**DOI:** 10.5888/pcd11.130261

**Published:** 2014-03-20

**Authors:** Nancy Amerson, Marguerite Nelson, Abigail Radcliffe, Conny Moody, Lori Williams, Cheryl Miles

**Affiliations:** Author Affiliations: Marguerite Nelson, Conny Moody, Cheryl Miles, Illinois Department of Public Health, Springfield, Illinois; Abigail Radcliffe, Lori Williams, Illinois Hospital Association, Springfield, Illinois.

## Abstract

Sodium reduction strategies on a population-based level are promoted as a public health intervention. Small and rural hospitals in Illinois were funded to adopt sodium reduction strategies as an intervention, have their hospital cafeteria and vending machines assessed via an environmental scan, and participate in an evaluation. Intervention strategies to identify and to label lower-sodium foods were implemented most, and pricing strategies were implemented least among funded hospitals. The sodium reduction strategies implemented could be replicated in other small and rural hospitals, because they were done with minimal funding and with minimal barriers.

## Objective

On a population-based level, policies to reduce sodium in foods served in institutional settings (eg, hospital cafeterias) are recommended to address high blood pressure (1). Approximately 65% of sodium consumed in 10 food categories comes from food obtained at a retail store (2). The hospital food environment affects patients, work site employees, and the larger community as families and visitors make food purchases in hospital facilities. With this project, we wanted to increase the adoption of sodium reduction in small and rural hospitals and to determine the factors associated with adoption of sodium reduction strategies.

## Methods

Using guidance from the Centers for Disease Control and Prevention (CDC), 9 of 88 small and rural Illinois hospitals received funding to increase adoption of sodium reduction strategies in the hospital setting. A Web-based survey was deployed at 2 points during the grant funding period to identify the sodium reduction strategies in place in small and rural hospitals. The first survey was deployed to funded hospitals to identify sodium reduction strategies in place at funded hospitals as of March 1, 2012. Funded hospitals were required to form a multidisciplinary team and select at least 1 intervention strategy from *Under Pressure: Strategies for Sodium Reduction in the Hospital Environment* (1) to implement during a 4-month period. A postintervention survey was deployed to identify sodium reduction strategies in place after the intervention among funded hospitals. The survey was also sent to all unfunded hospitals to assess what, if any, sodium reduction strategies were being used.

Illinois Cardiovascular Program staff worked with staff from CDC’s Division of Nutrition, Physical Activity, and Obesity to modify a Healthy Hospitals Environmental Scan (scan) for use in Illinois’s funded small and rural hospitals. The scan was originally modified by CDC from the Nutrition Environment Measures Survey (3,4). The scan consists of approximately 200 questions and 2 areas for assessment: the cafeteria and the vending machines. Additional questions were added to the scan to collect more sodium-specific data. The Illinois Hospital Association, with permission of the funded hospitals, conducted the prescan in April 2012 before the initiative and a postscan in August 2012 at the conclusion of the grant funding.

## Results

All 9 of the funded hospitals completed both surveys. An additional 21 unfunded hospitals completed a survey regarding sodium reduction activities (response rate: 21/79, 26.6%). Before the sodium reduction initiative, a combined total of 144 sodium reduction strategies were already in place at funded hospitals. Grant funding contributed to an additional 95 sodium reduction strategies being implemented among funded hospitals. The number of strategies added by each hospital ranged from 2 to 19. Table 1 highlights the differences in the number of sodium reduction strategies implemented before and after the initiative in funded hospitals. Each hospital implemented at least 1 intervention strategy from the required list.

**Table 1:** Sodium Reduction Strategies Before and After Initiative, Illinois, March to July 2012

Site	No. of Strategies in Use as of March 1, 2012	No. of Strategies in Use as of July 15, 2012	Difference
**Funded hospitals (n = 9)**			
Hospital A	5	11	+6
Hospital B	15	34	+19
Hospital C	8	26	+18
Hospital D	5	22	+17
Hospital E	36	45	+9
Hospital F	26	28	+2
Hospital G	7	19	+12
Hospital H^a^	21	27	+6
Hospital I^a^	21	27	+6
Total	144	239	+95
**Unfunded hospitals (n = 21)**	NA	337	—

Abbreviation: NA, not applicable.

a Two hospitals are part of the same health system; 1 survey was completed to represent strategies implemented at both hospitals.

On average, the funded hospitals were implementing 16 sodium reduction strategies per hospital before the initiative. At the conclusion, the average number of strategies per hospital increased to 27. Unfunded hospitals are implementing an average of 16 sodium reduction activities per hospital, identical to the funded sites pre-assessment. Table 2 highlights the strategies in place most often among funded hospitals after the intervention. Support from dietary councils and wellness programs from within and outside the hospital organization was cited most often as a facilitator for successful implementation. Administrative obstacles were cited most often as a barrier and included issues with contracts and timelines.

**Table 2:** Sodium Reduction Strategies Most Often Implemented Among Hospitals Funded by Initiative, Illinois, July 2012

Sodium Reduction Activity	Funded (n = 8^a^)		Unfunded, July 2012 (n = 21), %
	March 2012, %	July 2012, %	
Have a dialogue about the current food environment in your hospital and desire for improvements with a multidisciplinary team from the hospital.	75	100	43
Conduct an environmental scan of foods and beverages currently served and all settings in which foods and beverages are served.	63	100	48
Adopt language supporting the availability of healthful, lower-sodium food at meetings and workshops hosted by or at your hospital.	38	100	48
Include sodium information, especially major sources of sodium, as part of cardiac diet education to heart patients.	88	88	95
Place lower-sodium, more healthful options such as fruit at and around the point of purchase.	63	88	76
Label foods to identify those considered healthful.	25	88	52
Provide nutrition information in and around food service settings in the hospital (eg, table tents, signage, menu labeling, murals, brochures).	50	88	48
Establish a fast-food–free zone by disallowing outside fast-food chains to operate in your facility.	63	88	43
Prepare educational materials for patients’ families regarding the impact of sodium on blood pressure and hidden sources of sodium.	50	75	76
Incorporate nutrition information, including the importance of sodium reduction, into the hospital newsletter and other publications.	38	75	29
Partner with other community organizations on nutrition projects.	50	75	29
Communicate the business case for environmental change to hospital administrators and hospital boards of directors.	63	75	29
Assemble a core food team to assess the current hospital food environment, including what is currently available and attitudes and beliefs relating to food served in the hospital setting.	63	75	38
Display thought-provoking advertisements on vending machines and around food service settings, such as comparing a piece of fruit or 100% fruit juice with a traditional packaged snack and a message to “Choose wisely, your heart will thank you.”	25	75	14

a Two hospitals are part of the same health system; 1 survey was completed to represent strategies implemented at both hospitals.

The food environment of the funded small and rural hospitals changed over the course of the intervention. The scan results showed an increase in the availability of sodium information and sodium messages in the hospitals. The number of hospitals that provide sodium information in the cafeteria, in printed brochures, and vending machines increased. Easy access to discretionary salt use (eg, salt shakers or packets on tables) remained high in funded hospitals. However, we found little to no change in pricing strategies to promote healthful food items.

## Discussion

Most strategies that were implemented were from the *Under Pressure* guidance document. A few strategies were developed by the hospitals. Lower-sodium food options were promoted more in funded hospitals at postintervention. Funding allowed for an increase in the number of sodium reduction strategies addressed and allotted for time and effort to be directed toward increasing implementation. Messaging about healthful items increased in the cafeteria and vending machine environments.

Changing the food environment in the hospital cafeteria and vending machines by promoting lower-sodium foods can occur quickly. More effort may be needed to use pricing strategies to promote more healthful foods. This systems-level change may involve more stakeholders and require more time for it to be implemented.

The timeframe in which the intervention was implemented may have been a limitation. Funded sites were awarded in March 2012 and the intervention was completed in June 2012. The 4-month period may have limited the types of interventions attempted. Other project limitations include not examining the sodium content of meals, tracking sales of newly offered food products to monitor consumption, or evaluating whether hospitals added more lower-sodium products as choices rather than replace high-sodium products.

As a result of the sodium reduction initiative in small and rural hospitals, funded sites were able to make policy and system-level changes to affect their patients, hospital employees, and the community at large. The sodium reduction activities implemented through this initiative were done with minimal funding and with minimal barriers. These factors make replication of these activities promising in other small and rural hospitals.
